# Analysis of complex trophic networks reveals the signature of land-use intensification on soil communities in agroecosystems

**DOI:** 10.1038/s41598-021-97300-9

**Published:** 2021-09-14

**Authors:** Juliette M. G. Bloor, Sara Si-Moussi, Pierre Taberlet, Pascal Carrère, Mickaël Hedde

**Affiliations:** 1 Université Clermont Auvergne, INRAE, VetAgro-Sup, UREP, Clermont-Ferrand, France; 2 Eco&Sols, Université Montpellier, CIRAD, INRAE, Institut Agro, IRD, Montpellier, France; 3 Laboratoire d’Ecologie Alpine (LECA), CNRS, Université Grenoble Alpes, Grenoble, France; 4 UiT – The Arctic University of Norway, Tromsø Museum, Tromsø, Norway; 5 Laboratoire TIMC-IMAG, CNRS, Grenoble INP, Université Grenoble Alpes, Grenoble, France

**Keywords:** Ecology, Environmental sciences

## Abstract

Increasing evidence suggests that agricultural intensification is a threat to many groups of soil biota, but how the impacts of land-use intensity on soil organisms translate into changes in comprehensive soil interaction networks remains unclear. Here for the first time, we use environmental DNA to examine total soil multi-trophic diversity and food web structure for temperate agroecosystems along a gradient of land-use intensity. We tested for response patterns in key properties of the soil food webs in sixteen fields ranging from arable crops to grazed permanent grasslands as part of a long-term management experiment. We found that agricultural intensification drives reductions in trophic group diversity, although taxa richness remained unchanged. Intensification generally reduced the complexity and connectance of soil interaction networks and induced consistent changes in energy pathways, but the magnitude of management-induced changes depended on the variable considered. Average path length (an indicator of food web redundancy and resilience) did not respond to our management intensity gradient. Moreover, turnover of network structure showed little response to increasing management intensity. Our data demonstrates the importance of considering different facets of trophic networks for a clearer understanding of agriculture-biodiversity relationships, with implications for nature-based solutions and sustainable agriculture.

## Introduction

Soil is a key reservoir of biological diversity on Earth, supporting complex food webs and biotic interactions that underlie biogeochemical cycling and the provision of fundamental ecosystem services such as plant production, carbon storage and the biological regulation of pest species^1,2^. Indeed, biodiverse soils are considered to play a pivotal role in sustainable agricultural systems and food security^1,3^. In recent years, widespread concerns over global biodiversity loss in aboveground organisms have heightened the interest in soil biodiversity patterns in response to global change^4–6^. Nevertheless, holistic understanding of the impacts of global change pressures on the diversity and interactions within complex networks of soil organisms remains limited^7^.

Soil systems are particularly sensitive to changes in land-use and management practices which modify soil physico-chemical properties^6,8^. Agricultural intensification can affect soil biodiversity both directly (through the application of pesticides, fertilizers and tillage) and indirectly, via changes in plant diversity and production which affect inputs of organic matter to the soil^5,9,10^. A large body of research suggests that intensive agricultural management practices and mineral fertilizer inputs can have negative effects on the species richness of many soil functional groups including earthworms, springtails, nematodes and oribatid mites^9,11–13^. Increasing land-use intensity has also been associated with decreases in microbial biomass^10,14^ and/or shifts in the relative abundance of different microbial groups (bacterial, archaeal and fungal taxa)^15,16^. For example, increased inorganic nitrogen inputs and organic matter with a low C:N ratio are commonly reported to reduce the fungal:bacteria ratio in intensively-managed agroecosystems^10,16^. Such responses to agricultural intensification at the species- or functional group-level can have implications for soil food web structure and trophic networks by modifying trophic resource availability, and hence the potential for trophic interactions and the prevalence of highly-connected taxa. Indeed, recent evidence suggests that intensive land-use management decreases the complexity of soil bacterial networks^17^, as well as that of soil networks based on a relatively-limited number of micro-/mesofaunal groups^8,9^. However, agricultural intensification is not consistently harmful to soil fauna diversity^16,18,19^, and variation or non-linearity in species responses may therefore lead to idiosyncratic effects on complete food web structure. Given that loss of interactions among species may also supersede the effects of species loss within trophic groups on ecosystem function^20^, there is a pressing need for data on the responses of complex soil interaction networks to land-use intensification.

To date, the construction of comprehensive food webs and entire interaction networks for soil communities has encountered a number of technical difficulties. As soil organisms show a 10^6^-fold difference in both body size and abundance^2^, traditional taxonomic sampling methods show considerable variation in spatio-temporal resolution and are difficult to standardize across groups. In addition, simple co-occurrence analysis of soil organisms may not accurately reflect or represent trophic interactions^21^. Consequently the construction of interaction networks requires detailed information on the nature of interactions and predator–prey relationships between different taxonomic groups which may be difficult to acquire for complex communities^22^. Over the last decade, environmental DNA and metabarcoding technology (DNA taxonomy combined with high-throughput DNA sequencing) have emerged as a promising tool for soil biodiversity monitoring^23–25^. Environmental DNA (eDNA) refers to the DNA present in environmental samples (such as soil, water or air), and includes a mixture of genomic DNA from both living cells/organisms (e.g. from skin, mucus or secretions) as well as extracellular DNA linked to cell death and degradation. Compared to the traditional description of taxonomic-based trophic networks, eDNA allows a faster and efficient construction of more comprehensive networks based on presence data^23,26^. Moreover, the identification and assignment of trophic groups in complex networks now benefits from the increasing availability of open knowledge bases on biotic interactions^27^ and functional guilds^28^. It is therefore possible to combine inherently-qualitative eDNA data with information on trophic interactions, and overcome some of the traditional limitations of soil food web analysis. However, although eDNA metabarcoding has clear potential to provide novel insights into the drivers of changes in soil biotic communities, this methodology has rarely been tested in the context of agricultural intensification^29^.

**Figure 1:**
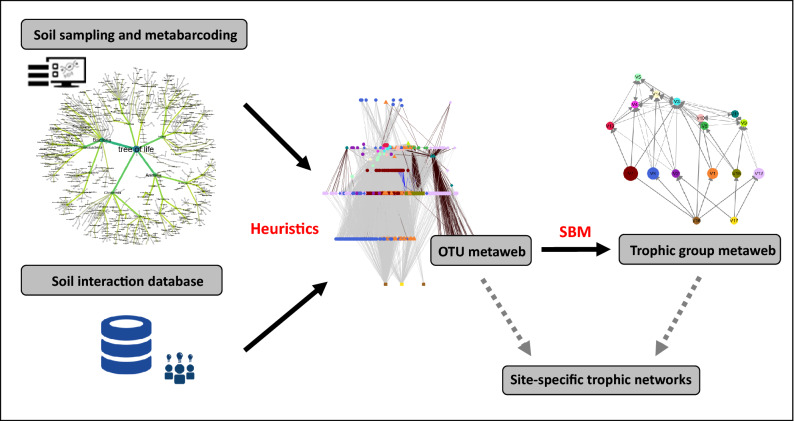
Inference of trophic network structure for the present study using Next‐Generation Sequencing technologies and a database of ecological interactions.

Here we use eDNA metabarcoding, data mining on trophic interactions and machine-learning in order to infer comprehensive, interaction-based soil food webs (Fig. 1), and to examine the trophic structure of soil communities for sixteen agroecosystems along a gradient of agricultural pressure (from arable crops to grazed permanent grasslands) in a long-term management trial. Analyses of food web properties and trophic networks were used to assess the potential for different network metrics to discriminate between agricultural practices, and to determine the sensitivity of trophic networks to land-use intensity. Our overarching hypothesis was that land-use intensification would decrease the trophic complexity of soil communities. Specifically, we hypothesized that increasing land-use intensity would reduce trophic network size, modifying soil food web connectance (number of realized interactions) and average centrality (prevalence of highly-connected species). In addition, we examined impacts of increasing land-use intensity on energy-flow pathways within trophic networks, predicting an increase in the dominance of bacteria over fungi but a decrease in the importance of parasitic interactions (the latter response due to a decrease in trophic resources). Finally, we explored the linkages between multi-trophic β-diversity (i.e. turnover in trophic group networks between fields) and land-use intensification, in order to test for possible divergence between the responses of local assemblages (α-diversity) and compositional turnover to agricultural pressure. Previous work on a limited number of soil trophic groups has suggested that this phenomenon occurs in temperate grassland communities along a land-use intensification gradient^19^. Such empirical evidence could demonstrate the role of biotic interactions in determining the outcome of ecosystem responses to land-use intensification, and highlight which network metrics may be useful indicators of soil biodiversity in managed ecosystems.

## Results

### Taxonomic composition and trophic groups

Metabarcoding data yielded a final database (and metaweb) comprised of 21,667 pairwise interactions (6% parasitic, 0.5% symbiotic), covering 99.7% of the taxa recorded (total of 1049 families/species/genera). 981 taxa were retained after filtering marine organisms and taxa from non-temperate terrestrial ecosystems (Supplementary Data table). Assessment of the taxonomic diversity among all fields showed that 14%, 48% and 38% of OTUs were assigned to family, genus and species level respectively. Across all fields, five out of 44 phyla represented about 50% of all taxa. The Proteobacteria were the most represented phylum (17.1% of the taxonomically-identified sequences), followed by Arthropoda, Actinobacteria, Nematoda and Ciliophora comprising 9.1%, 7.7%, 7.7% and 6.5% of all taxa respectively. On average we detected 369 taxa in each experimental field. Overall, 79.1% of phyla and 36% of families were found to be common across all experimental fields. Trophic inference generated a total of 16 trophic groups across experimental fields (range 12–16 per field, Supplementary Fig. S1).

### Food web responses to land-use intensification

**Figure 2:**
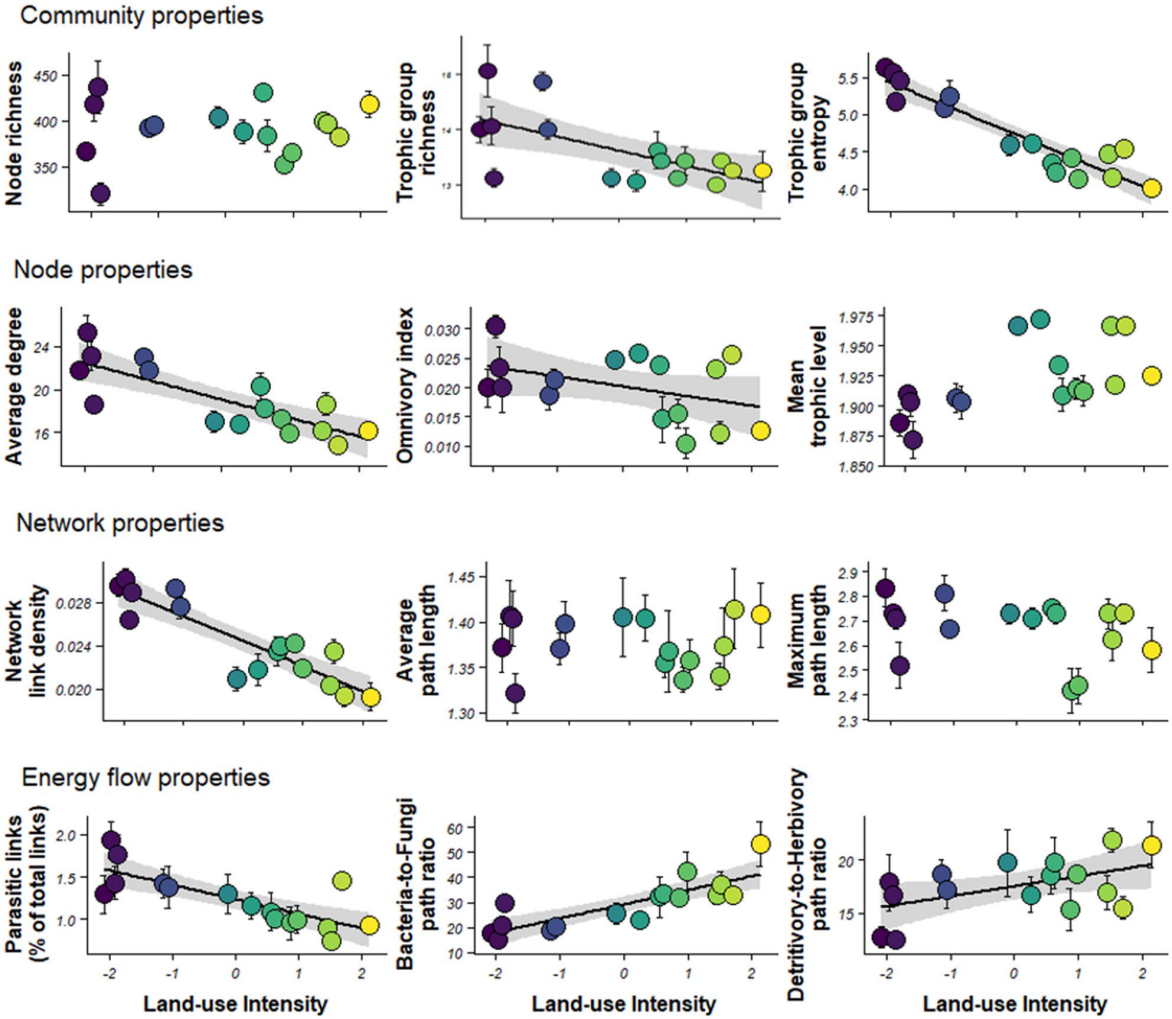
Relationships between land-use intensity and average trophic network properties across experimental fields. Regression lines and 95% CI are shown where significant. Network metrics are given in Table 2. The colour code emphasizes the land-use intensity gradient from least intensive (purple) to most intensive (yellow).

Assessment of food web properties indicated considerable variation in diversity and structure across experimental fields (Fig. 2). Eight out of the 12 network indices representing α-diversity and network topology showed significant linear responses to agricultural intensification gradient (Fig. 2, Supplementary Table S1). Total number of trophic groups and trophic group equitability (entropy) declined along the gradient of intensification, as did average node degree, omnivory level, link density and prevalence of parasitic links. The magnitude of decrease in response to increasing land-use intensity was greatest for average degree, trophic group entropy, network link density and parasitic links (standardized effect sizes, SES, ranging from − 0.15 to − 0.22); decreases in trophic group richness and omnivory index were more limited (SES − 0.03 and − 0.10 respectively). In contrast, both the bacteria-to-fungi path ratio and the detritivory-to-root herbivory path ratio increased along the gradient of intensification, with a two-fold increase from values in ‘low-intensity’ fields to those at the ‘high-intensity’ end of the gradient (SES of 0.16 and 0.09 for the bacteria-to-fungi path ratio and the detritivory-to-root herbivory path ratio respectively). Total number of taxa (node richness), mean trophic level, mean and maximum path length showed no response to the agricultural intensification gradient (Fig. 2).

**Figure 3:**
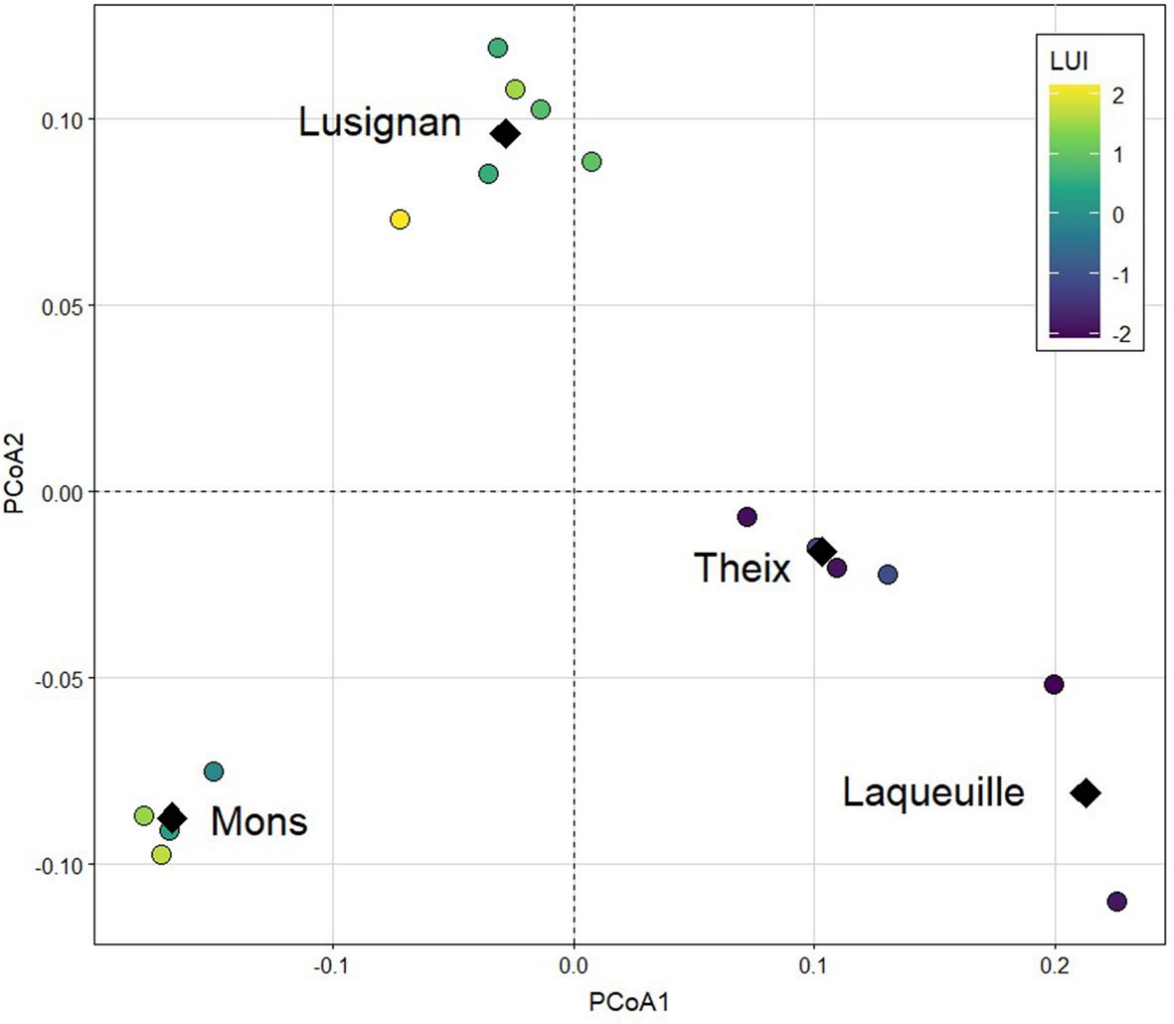
Principal Coordinates Analysis based on food web dissimilarities for interaction networks constructed with Operational Taxonomic Units (OTUs) in experimental fields. Colours illustrate the gradient of land-use intensity (LUI) across experimental fields. Black diamonds represent the barycenter of each study sites.

Analysis of network dissimilarity across experimental fields indicated a significant turnover in node composition between fields (DF = 15, F = 10.08, *p* < 0.01) and sites (DF = 3, F = 3.33, *p* < 0.01) for taxa-based food webs (Fig. 3), but no significant turnover in trophic group networks. Permanent grassland fields at the low end of the land-use intensity gradient were characterized by greater β-diversity compared to the more-intensively managed fields at the Mons and Lusignan sites (Fig. 3), but network dissimilarity showed no clear relationship with land use intensity (LUI). Site rankings along PCoA1 were negatively correlated to soil pH across experimental fields (r = − 0.96, *p* < 0.05), but were unrelated to any other pedoclimatic variables.

## Discussion

The construction of data-based food webs including multiple interaction types is essential for the accurate appraisal of ecosystem vulnerability to global change^23,30,31^. Recent work on complex aboveground trophic networks has shown that agricultural intensification drives changes in network structure and robustness^32^, but studies of complex soil networks are lacking and current understanding of soil network responses to land-use is based on a relatively-limited number of taxonomic groups and network metrics^8,9^. Our study of complex soil food webs generated by eDNA metabarcoding in 16 agricultural fields along a broad LUI gradient resulted in three major findings: (1) trophic interaction networks show a range of consistent qualitative responses to agricultural intensification; (2) soil network complexity is more sensitive to agricultural intensification than network size; (3) local assemblages (metrics of α-diversity) show stronger statistical relationships with agricultural intensification than does composition turnover (β-diversity). Together our results bring new insights into the importance of different facets of food web structure for biodiversity preservation, and the contribution of biological interactions to agriculture-biodiversity relationships.

Five out of nine network metrics describing node, community and network properties showed significant negative relationships with land-use intensity in our study, providing support for the hypothesis that increasing management intensity decreases trophic diversity and the complexity of soil interaction networks. In general, we found that the magnitude of negative effects induced by our LUI gradient was greater for soil network complexity than network size; agricultural intensification was associated with markedly-simpler networks (lower interactions and distribution of interactions between nodes) compared to relatively-limited changes observed in trophic group richness. Such a loss of network complexity and cohesion has previously only been demonstrated for soil microbial networks in relation to cropping intensity^17,33^, although our observed negative relationship between link density and LUI also matches results from comparisons between soil networks in grasslands and arable crop systems^8^. Reduced diversity of interactions may stem from less structurally-complex habitats, and shifts in environmental and/or biotic filtering under intensive management practices^34,35^. Decreases in trophic network complexity are considered to have repercussions for community stability and soil functioning in a changing environment^31^, and recent work with combined above- and belowground trophic groups has also demonstrated linkages between network complexity and the provision of ecosystem services^36^.

We found greater effects of LUI on trophic group richness/equitability compared with taxa richness, in line with the idea that species number is less critical for soil ecosystem function than functional diversity due to functional redundancy in soils^37^. Where newly-occurring or “lost” species exhibit unique functional attributes, changes in functional richness can markedly exceed the change in taxonomic diversity. In systems with relatively low functional redundancy, increases in LUI may therefore cause strong functional shifts. Limited responses of taxa richness to LUI observed in the present work could partly reflect the dominance of smaller-bodied organisms in our food webs, since previous studies have suggested that larger soil organisms and top predators show greater sensitivity to agricultural intensification compared with smaller-sized soil biota^7^. Low rates of occurrence of large top predators in our dataset may also contribute to the limited responses of average path length and mean trophic level to agricultural intensification in our soil networks; additional studies with biodiversity ‘soups’ and complementary sampling methods could help test the generality of our findings^38^.

Energy-flow pathways within trophic networks reflect the quantity and quality of resources, stochiometric constraints and ‘top-down’ regulatory processes^39,40^. In the present study, increasing management intensity increased the relative importance of both the bacterial energy channel compared to the fungal channel, and the detrital energy channel compared to the root herbivory channel. These results are consistent with changes in plant biomass allocation and rates of decomposition, root exudation and nitrogen mineralization under agricultural intensification, which promote the detrital energy channel^39,41^, but see^10^. Moreover, the ‘slow cycle’ fungal energy channel tends to be more dominant in extensively-managed systems where the ratio of carbon to nitrogen (C:N) in organic matter is high. Although some interconnections between energy channels exist^42^, dominance of fungal, bacterial or root energy channels in soil trophic networks has significant implications for subsequent nutrient turnover rates and ecosystem stability^39^. Our results highlighted a decrease in the prevalence of parasitic interactions with increasing agricultural pressure. This response pattern likely reflects a decrease in trophic resources (vector abundance) due to the increased use of pesticides and fertilizers along our LUI gradient. Data on soil parasites and agricultural intensification is scarce in the literature, but decreases in the prevalence of parasitic nematodes and mites have previously been observed following the conversion of grassland to arable land^12^.

In contrast to our metrics of network size and structure or energy channels, turnover in trophic networks (β-diversity of nodes) showed no clear pattern with increasing LUI across our study systems. Moreover, we found no significant turnover in trophic groups, supporting the idea of a common “backbone” of interactions across food webs^43^. These results suggest that agricultural intensification has divergent effects on local interaction networks and the network turnover of soil food webs across different sites, with greater effects of LUI on α- rather than β-diversity. Indeed, the marked responses of local assemblages (α-diversity) to agricultural intensification in the present study could have served as a stabilizing mechanism for broader-scale patterns of diversity^44^. Limited β-diversity responses and absence of biotic homogenization to LUI may also be linked to confounding effects of pedoclimatic conditions^15,45^, or to functional redundancy and phylogenetic niche conservatism^46^. Irrespective of the underlying mechanisms, insensitivity of beta diversity to increasing agricultural pressures may promote the buffering capacity of soil food webs against land-use change, with cascading effects on the connections between detritus-based and primary production-based food webs, and the regulation of aboveground and belowground dynamics^40^.

With regards to our methodological approach, eDNA metabarcoding can meet challenges faced by traditional taxonomic monitoring, use high taxonomic resolution to confront current paradigms of ecosystem dynamics, and provide a valuable tool to support evidence-based decision making^47^. In the present study we show that eDNA metabarcoding is an effective technique for visualizing complex soil communities, and for analysing the structural and functional relationships within soil food webs. To date, very few studies on soil food web structure integrating microbes, micro- and mesofauna have documented large numbers of taxa (> 500), and those rare examples involved spatially-distinct and labour-intensive diversity protocols^8^. Not only were we able to identify a very high number of taxa from microbes and key micro- and meso-faunal groups using a simple field protocol, but the analysis of standard soil samples also provided good assurance of species/ taxa co-occurrence in space.

Unlike bulk-community DNA sampling which targets whole organisms, eDNA samples detect biological signals in the environment from traces of intracellular and extracellular DNA. Given that DNA fragments may persist for periods ranging from several days to several months in the environment, this increases the temporal uncertainty associated with the diversity assessment^47^. It is therefore possible that taxa detected in soil samples in the present study were not all present at exactly the same time, and hence our food webs represent the potential complexity of trophic interactions in our experimental fields, rather than a snapshot of trophic interactions at a single date. Further work using additional sampling dates, and combined bulk- and eDNA sampling, would provide a clearer picture of the degree of temporal uncertainty associated with eDNA metabarcoding results at our study sites.

Finally, quantifying land-use intensity is a non-trivial issue in studies of agricultural intensification, since multiple processes relating to inputs, outputs and mechanical disturbance need to be considered^48,49^. As yet, there is no widely-accepted, purely quantitative measure for ranking a set of agricultural fields along a gradient of management intensity, although indices of LUI do exist for functionally-similar agroecosystems such as grasslands^19,48^ or arable cropping systems^33^. In the present study, we used an unweighted, multicriteria approach^48^ and calculated a continuous LUI index based on management practices over the preceding 6-year period. Our approach and choice of criteria allowed the positioning of very different agroecosystems (permanent grasslands, annual cropping systems) along a standardised gradient, and the site ranking obtained was subsequently validated by expert opinion (ANAEE-F ACBB site managers). Our calculation of the intensification gradient also provided an objective method to quantify the differences in agricultural intensification between sites, which is not possible to achieve based on expert knowledge alone. Of course, the absolute values of such an index will change depending on the exact criteria used and the cohort of sites examined, making direct comparisons between studies difficult. The development and validation of a standardised, additive index for use across the entire range of cropping systems and managed grasslands would facilitate the identification of broad-scale response patterns and assessment of the relative importance of drivers of agricultural intensification in future studies.

## Conclusion

Together, the results of this study extend previous work on multiple taxa and land-use, and highlight the need to consider the response patterns of complex trophic networks for a clearer understanding of linkages between biodiversity and agricultural practices. Our results indicate that agricultural intensification has profound impacts on trophic interactions and soil trophic network structure, driving decreases in trophic group diversity (richness, equitability) and complexity of interactions (average degree, link density). Observed changes in energy flow pathways were consistent with shifts in resource availability (labile C, living roots) and increases in pesticide use due to agricultural intensification. In contrast, eDNA metabarcoding did not highlight sensitivity to agricultural intensification for either taxa richness, maximum path length or mean trophic level. Soil trophic networks in our studied agroecosystems showed divergent responses of α- and β-diversity to intensification, and we also found evidence for a common backbone of trophic group interactions across food webs, with implications for both ecological assembly rules and diversity conservation strategies. Although more broad-scale studies which include the temporal dynamics of complex soil food webs are needed, the present results reveal that agricultural intensification simplifies entire soil trophic networks, with likely consequences for biodiversity-ecosystem function relationships.

## Methods

### Field sites and sampling

Sixteen experimental fields were selected from within four sites of the long-term ANAEE-F ACBB agroecosystems management trial in France (http://www.soere-acbb.com/): Mons (49° 52′ N, 3° 1′ E, 85 m a.s.l.), Lusignan (46° 25′ N, 0° 7′ E, 151 m a.s.l.), Theix (45° 43′ N, 3° 1′ E, 880 m a.s.l.) and Laqueuille (45° 38′ N, 2° 44′ E, 1100 m a.s.l.). These four temperate sites cover a range of soil types and climate conditions, and represent a gradient of management intensity from permanent grasslands to arable cropping systems (Table 1). At each site, we selected fields in order to generate a broad gradient of management intensity across sites (Table 1); at the time of the study, fields had been subjected to experimental treatments for over 10 years. The gradient of management intensity for the 16 fields was quantified using a multi-criteria evaluation based on soil tillage (depth, frequency), N inputs (mineral N, N fixation), C exports (biomass harvests, residue management) and frequency of crop protection treatments for the period 2010–2016; fields were ranked according to their scores from the first axis of a principal components analysis using the management criteria (Supplementary Fig. S2).

**Table 1:** Soil type, management and land-use intensity (LUI) ranking for the sixteen experimental fields in France.

Site	Soil type	Agroecosystem	Management	LUI
Mons	Silty loam (Luvisol)	Arable crops (6-years rotation: spring pea; winter wheat; rapeseed; spring barley; maize; winter wheat)	Conventional tillage, reference N inputs	1.70
			Conventional tillage, reduced N inputs	1.46
			Reduced tillage	-0.11
			Reduced tillage, residue removal	0.26
Lusignan	Loamy clay (Cambisol)	Arable crops (3-years rotation: maize; wheat; barley)	Conventional tillage, reference N inputs	2.14
		Crop-grass rotation (6-years rotation: maize; wheat; barley; 3-years sown grassland)	Tillage for crops, mown grassland, reference N inputs	1.52
		Grass-crop rotation (9-years rotation: 6-years sown grassland; maize; wheat; barley)	Tillage for crops, mown grassland, reference N inputs (2 fields, one with additional pesticide treatments)	0.98
				0.87
			Tillage for crops, mown grassland, reduced N inputs	0.62
		Grass-crop rotation (9-years rotation: 6-years sown grassland with legumes; maize; wheat; barley)	Tillage for crops, Grazed grassland	0.57
Theix	Sandy clay loam (Cambisol)	Mown permanent grassland	Reference NPK inputs	− 1.05
			No fertilizer inputs	− 1.91
	Clay loam (Cambisol)	Mown permanent grassland	Reference NPK inputs	− 1.13
			No fertilizer inputs	− 1.96
Laqueuille	Silt loam (Andosol)	Grazed permanent grassland	High stocking rate, Reference N inputs	− 1.86
			Low stocking rate, no N inputs	− 2.08

Values for LUI are based on a multicriteria evaluation (Supplementary Fig. S2); more positive values indicate more intensive management practices. Mean annual temperature and rainfall of sites as follows: Mons: 11.7 °C, 614 mm; Lusignan: 11.7 °C, 800 mm; Theix: 8.7 °C, 780 mm; Laqueuille: 8 °C, 1000 mm.

Soil sampling was carried out at a standardized plant phenological stage across sites i.e. the peak of the plant vegetative growing period in 2016 (mid-May to mid-June depending on the plant phenology at each site). Four plots of 0.25 m^2^ were selected for sampling in each experimental field; plots were randomly-positioned in each quadrant of the field, at least 10 m apart and at least 5 m away from the edge of the field. One soil core (10 cm depth, 8 cm diameter) was taken from each plot and coarsely-sieved to remove stones/large debris (heat-sterilized sieve, 4 mm mesh size), providing a total of 64 soil cores. A 15 g sample of freshly-sieved soil from each soil core was placed in a sterile plastic container and preserved with silica gel within 30 min of field collection to prevent microbial growth prior to DNA extraction^50^.

### Molecular analysis and sequence curation

Soil biodiversity was assessed with DNA metabarcoding using eight DNA markers. Three markers targeted regions of the ssu rRNA gene in the Bacteria and Eukaryota domains, whereas five additional markers focused on earthworms, collembola, oligochaetes, insects and vascular plants (primer details for Bact02, Coll01, Euk02, Euk03, Inse01, Lumb01, Oligo01, Sper01 given in Supplementary Table S2). Extracellular DNA was extracted from 15 g of soil following methods described previously^51^. Briefly, soil was extracted with a saturated phosphate buffer (Na_2_HPO_4_; 0.12 M; pH 8); sub-samples of the slurry were centrifuged, and the resulting supernatant was used as starting material for the NucleoSpin Soil kit (Macherey–Nagel, Düren, Germany), following manufacturer’s instructions but skipping the lysis step. The resulting DNA extracts were recovered in 100 µL and diluted ten times. Each diluted extract was then PCR-amplified, with all the above primer pairs. All PCR were carried out in a final volume of 20 μL containing 2 μL of DNA extract. The amplification mixture consisted of 10µL of AmpliTaq Gold 360 master mix (Applied Biosystems), 0.5 μM of each primer and 0.16 µL (20 mg/mL) of bovine serum albumin (BSA, Roche Diagnostic). Polymerase activation was performed at 95 °C for 10 min, followed by 32–45 cycles at 95 °C for 30 s (denaturation), 45–55 °C for 30 s (primer annealing) and 72 °C for 60–90 s (extension), followed by a final elongation for 7 min at 72 °C.

We carried-out four technical PCR replicates for each sample and for each primer pair. In order to minimize possible biases in the experimental workflow, each technical replicate for a primer pair included three extraction controls, 12 blanks (no primer, no template), nine PCR negative controls (ultrapure water), and eight positive controls (positive controls only for Sper01 and Euka02 primer pairs). PCR products were purified using the MinElute PCR purification kit (Qiagen GmbH). Library preparation was performed using the MetaFast protocol by Fasteris (https://www.fasteris.com/dna/?q=content/metafast‐protocol‐amplicon‐metagenomic‐analysis), which significantly limits the tag‐jump problem^50^. For Bact02, sequencing was performed by 2 × 250‐bp paired‐end sequencing on the Illumina MiSeq platform, while for all other primer pairs sequencing was performed by 2 × 125‐bp paired‐end sequencing on the Illumina HiSeq 2500 platform using default settings at Fasteris. Sequence data were processed using OBITools software^52^ to (1) assemble and dereplicate reads, (2) match sequences to the original samples, (3) remove noise from the data by removing singletons, low-quality sequences, putative PCR/sequencing artefacts (criteria used to remove low-quality reads^53^), and (4) taxonomically-assign the remaining sequences. At the end of the data curation process we obtained a total of 5880 robust OTUs (Operational Taxonomic Units).

### Construction of trophic interaction networks and inference of trophic groups

Heuristic food webs were constructed from DNA-generated taxa lists^54^; we identified all possible pair-wise trophic interactions using existing structured trophic knowledge, information mined from the literature and a rule-based approach (Fig. 1). We first determined the complete taxonomic tree for the OTUs using DNA reference databases and the Name Parser API of the Global Biodiversity Information Facility (GBIF)^55^. We next created a database of pairwise biotic interactions (predation, parasitism, symbiosis) for our complete taxonomic tree by compiling information from available databases, including NemaGuild, FunGuild^28^, BETSI (http://betsi.cesab.org/), GloBI^27^ and systematic literature searches. In a limited number of cases where data was lacking, we generalized the information on interactions within families. For example, in the absence of available data on species-level interactions, members of a fungivorous genus of nematodes were assumed to feed on all fungal taxa in the network. The database was trimmed based on species co-occurrence and habitat data; pairwise ‘resource-consumer’ interactions were only included for organisms known to co-occur in a soil layer (soil surface, 0–10 cm, > 10 cm). The database of plant and animal taxa was also trimmed based on geographical range, targeting taxa known to occur in temperate Europe (taxa of microorganisms were considered to have a broad geographical distribution). Local food webs for each plot were extracted from the metaweb based on the list of taxa present in the four sets of DNA sequences for each plot.

Given that many taxa share the same sets of resources and consumers, partitioning food webs into trophic groups can be a useful tool to better identify network structure and function^56,57^. Aggregating taxa into trophic groups allows the simplification of complex food webs whilst preserving the information content of the initial network structure. With this approach, information on intra-specific plasticity is lost, but we assume that inter-specific plasticity is greater than intraspecific plasticity and drives network structure responses at the trophic group level. We assigned taxa to trophic groups using an automatic inference technique and machine-learning approach to avoid limitations of subjective, user-defined groups^58^; stochastic block models (SBM)^59^ were fitted to the heuristic metaweb using the “blockmodels” package in R (Supplementary Fig. S3). This approach generates trophic groups based on a common resource/food source or predator irrespective of taxonomic affiliation (for example, nematodes and protists which feed on the same bacterial groups will be clustered together). The optimal number of groups was determined using the Integrated Complete-data Likelihood criterion (ICL)^60^. In addition to partitioning taxa into groups, the stochastic block model ‘learns’ the probability of interactions between trophic groups that generate the trophic group metaweb. Heuristic food webs based on DNA metabarcoding have previously been validated as a powerful tool in freshwater systems^61^.

### Statistical and network analyses

Standard metrics in connection with α-diversity and network robustness were used to describe the topology and properties of the 64 food webs inferred in the 16 experimental fields (Table 2, see Supplementary Fig. S4 for correlations between indices); in particular we focused on trophic network size, diversity of taxa or trophic groups (α-diversity), connectance, centrality and the nature of energy flow pathways (an indicator of network functioning) using the R package *Netindices*. In addition, we assessed multi-trophic β-diversity using the Simpson dissimilarity index and calculations of pairwise network dissimilarity between the 16 experimental fields. We computed the turnover in the composition (nodes) of both the trophic group networks and the taxa-based food webs using Hill numbers^62^) and the R package *econetwork*. We then applied generalized linear mixed effect models (GLMM) to test whether individually, these characteristics differed along our experimental land-use intensity gradient. Land-use intensity score and site identifier were used as fixed and random effects respectively:

$$m_{i} \sim LUI_{i} + { 1}|site_{i}$$

**Table 2:** Network metrics applied at experimental fields to assess soil biodiversity, trophic complexity and energy pathways of the soil food web^63,64^.

Metric	Scope	Definition	Biological relevance
Node richness	Community	Number of nodes $S$	Food web size. Indicator of potential relationships
Trophic group richness	Community	Number of distinct trophic groups present in a community.$|G|$	Functional diversity
Trophic group entropy	Community	Distribution of read counts among groups in an observed community using Hill numbers framework	Indicator of equitability
Average degree	Node	Number of interactions in which the OTU or group is involved	Centrality index: indicator of complexity
Mean trophic level	Node	Ordinal classification based on the relative position in the network, defined as 1 + the weighted average of the trophic levels of its resources/hosts	Centrality index: Indicator of the variety of trophic levels/ resources exploited as a food source
Omnivory level	Node	Variety in the trophic levels of a consumer’s food	Centrality index: Indicator of the degree of specialization
Link density	Network	Average number of edges per node L/S	Connectivity index: indicator of quantity of relationships
Average path length	Network	Mean distance (d) between any pair of nodes in the network Σ_i,j_ d_ij_/S^2^	Connectivity index: indicator of trophic redundancy and food web resilience
Maximum path length	Network	Longest path between any pair of nodes in the network max┬((i,j) ∈ 1..S)〖d_ij 〗	Connectivity index: indicator of network length
% parasitic links	Energy pathways	Proportion of total links that are parasitic	Diversity of interaction types
Bacteria:Fungi path ratio	Energy pathways	Ratio of decomposer-bacteria chains to saprotroph-fungi total pathway lengths	Indicator of bacterial dominance and N cycling efficiency
Detritivore:Herbivore path ratio	Energy pathways	Ratio between detritivore and root herbivory total pathway lengths	Indicator of importance of the detrital food path (dead vs. living organisms as a resource)
Node turnover (hill number: q = 0, 1,2)	Network stability	Turnover in the composition of the network (OTU or group)	β-diversity

The interaction network is described by nodes (N), which are either OTUs or trophic groups (G), and edges (L, links from a resource node to a consumer node). Edges are described by their type (predator, parasitic, symbiotic), and weighted by the probability of occurrence of the resource and consumer pair.

To visualize the overall effects of land-use intensity on β-diversity, we computed the first two components of the principal coordinate analysis (pcoa function from package *ape*) on the matrix of plot-by-plot network dissimilarities (Hill number = 0).

## Supplementary Information

Supplementary Information 1.

Supplementary Information 2.

## Data Availability

The complete list of taxa and associated trophic groups is provided as a data file. The datasets generated during and/or analysed during the current study are available on request. All soil samples were collected with permission from ANAEE-F ACBB site managers, and in accordance with ANAEE-F ACBB guidelines. No plant parts were used in the present study.
