# Supplementary material for: Analysis of complex trophic networks reveals the signature of land-use intensification on soil communities in agroecosystems
Source: Sci Rep. 2021 Sep 14;11:18260. doi: 10.1038/s41598-021-97300-9 (PMC8440573; doi:10.1038/s41598-021-97300-9)

**Analysis of complex trophic networks reveals the signature of land-use intensification on soil communities in agroecosystems**

**Supplementary Material**

Juliette M.G. Bloor^1*^, Sara Si-Moussi^2,3,5^, Pierre Taberlet^3,4^, Pascal Carrère^1^, Mickaël Hedde^2^

^1^Université Clermont Auvergne, INRAE, VetAgro-Sup, UREP, Clermont-Ferrand, France

^2^ Eco&Sols, Univ Montpellier, CIRAD, INRAE, Institut Agro, IRD, Montpellier, France

^3^Laboratoire d’Ecologie Alpine (LECA), CNRS, Université Grenoble Alpes, Grenoble, France

^4^ UiT – The Arctic University of Norway, Tromsø Museum, Tromsø, Norway

^5^Laboratoire TIMC-IMAG, CNRS, Grenoble INP, Université Grenoble Alpes, Grenoble, France

**Corresponding author*:** juliette.bloor@inrae.fr; ORCID 0000-0002-8668-1323

Tel.: +33-4437-616-02; Fax: +33-4437-616-29

**Table S1.** Results of linear mixed-model analysis for the effect of land-use intensity on all food web properties. Model parameters (and associated estimate uncertainties) are presented (n = 64, DF = 1).

| **Food web properties** | **Land-use intensity** | |  | **Intercept** | |  |
| --- | --- | --- | --- | --- | --- | --- |
| Node richness | < 0.001 | (0.015) |  | 5.91*** | (0.039) | |
| Trophic group richness | -0.01** | (0.004) |  | 2.66*** | (0.011) | |
| Trophic group entropy | -0.03*** | (0.007) |  | 1.76*** | (0.025) | |
| Average degree | -0.07*** | (0.153) |  | 2.97*** | (0.033) | |
| Mean trophic level | < 0.001 | (0.001) |  | 1.07*** | (0.006) | |
| Omnivory level | -0.002** | (<0.001) |  | 0.020*** | (0.002) | |
| Density | -0.002*** | (<0.001) |  | 0.025*** | (<0.001) | |
| Average path length | -0.003 | (0.002) |  | 0.86*** | (0.003) | |
| Maximum path length | -0.004 | (0.006) |  | 1.30*** | (0.009) | |
| % Parasitic links | -0.08*** | (0.014) |  | 0.75*** | (0.020) | |
| Bacteria:Fungi path ratio | 0.18*** | (0.039) |  | 3.38*** | (0.070) | |
| Detritivore:Herbivore path ratio | 0.05*** | (0.030) |  | 2.97*** | (0.056) | |

*** <0.001; ** <0.01; *<0.05

**Table S2.** DNA markers and associated PCR details used in molecular analysis.

| Code | forward primer, 5'-3' reverse primer, 5'-3' | Number of PCR cycles | Annealing temperature | Elongation time |
| --- | --- | --- | --- | --- |
| Bact02 | GCCAGCMGCCGCGGTAA GGACTACCMGGGTATCTAA | 32 | 53°C | 1'30" |
| Euka02 | TTTGTCTGSTTAATTSCG CACAGACCTGTTATTGC | 40 | 45°C | 1' |
| Euka03 | CCCTTTGTACACACCGCC CTTCYGCAGGTTCACCTAC | 40 | 55°C | 1' |
| Sper01 | GGGCAATCCTGAGCCAA CCATTGAGTCTCTGCACCTATC | 40 | 52°C | 1' |
| Olig01 | CAAGAAGACCCTATAGAGCTT CCTGTTATCCCTAAGGTARCT | 45 | 55°C | 1' |
| Lumb01 | CAAGAAGACCCTATAGAGCTT GGTCGCCCCAACCGAAT | 45 | 55°C | 1' |
| Coll01 | ACGCTGTTATCCCTWAGG GACGATAAGACCCTWTAGA | 45 | 51°C | 1' |
| Inse01 | RGACGAGAAGACCCTATARA ACGCTGTTATCCCTAARGTA | 45 | 52°C | 1' |

**Figure S1.** Distribution of kingdoms / resource types within the 16 trophic groups identified across experimental fields.


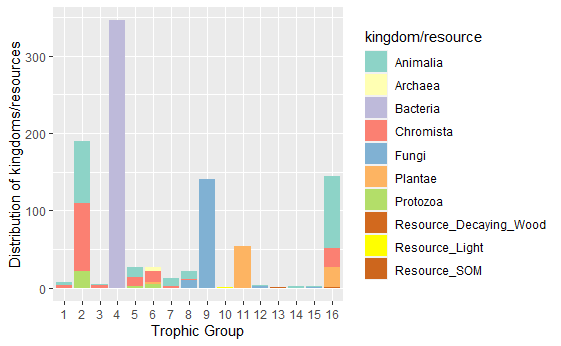


**Figure S2.** PCA biplot based on soil tillage (depth, frequency), N inputs (inorganic N fertilizer, N fixation), C exports (biomass harvests, residue management) and frequency of crop protection treatments in experimental fields. Symbols represent sites: triangles, Mons; squares, Lusignan; circles, Theix; diamonds, Laqueuille. Treatment codes are given by: T, conventional tillage; RT+RR, reduced tillage with residue removal; M, mown grassland; G, grazed grassland; +N, mineral N addition; -N, no mineral N or reduced mineral N inputs. Scores from the first axis are used to rank fields in terms of land-use intensity.


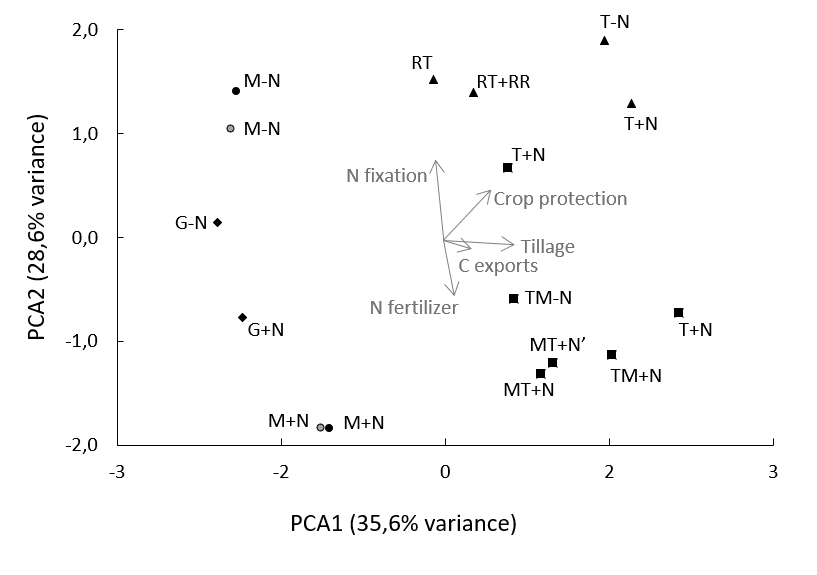


**Figure S3** Independent Completed Likelihood curve for the selection of optimal number of trophic groups. On the x-axis, the number of groups Q, on the y-axis the ICL score for each possible partition (black and red circles) into Q groups. The optimal partition for each value of Q is reported in red.


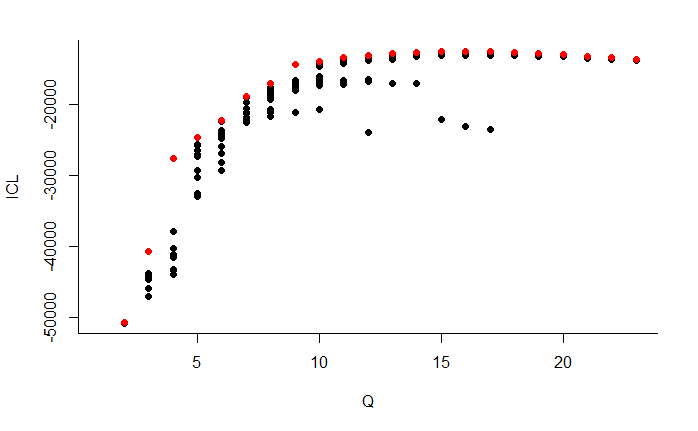


**Figure S4.** Heatmap of correlations between network metrics used to describe the soil food webs over a gradient of agricultural land-use.


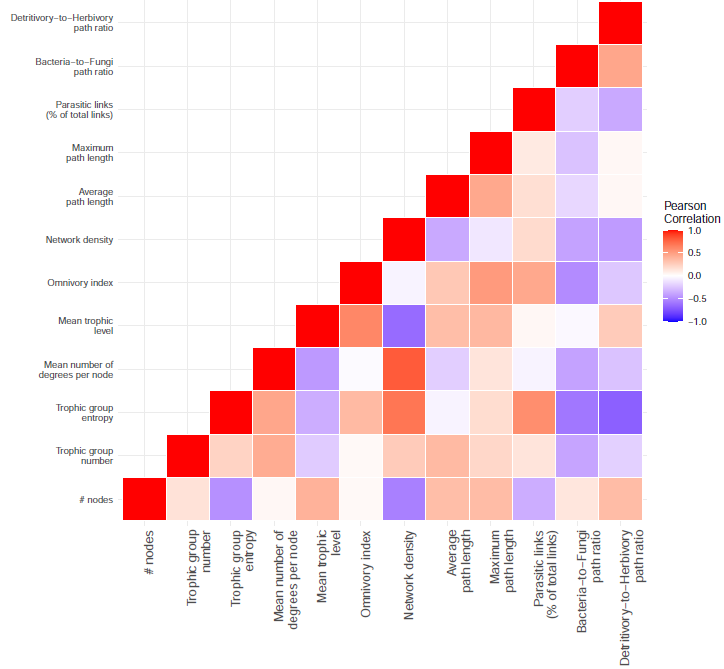

Supplement: Supplementary file 2 — Supplementary Information 2. [file 41598_2021_97300_MOESM2_ESM.docx]
